# Differential Impact of Tetratricopeptide Repeat Proteins on the Steroid Hormone Receptors

**DOI:** 10.1371/journal.pone.0011717

**Published:** 2010-07-22

**Authors:** Jan-Philip Schülke, Gabriela Monika Wochnik, Isabelle Lang-Rollin, Nils Christian Gassen, Regina Theresia Knapp, Barbara Berning, Alexander Yassouridis, Theo Rein

**Affiliations:** 1 Chaperone Research Group, Max Planck Institute of Psychiatry, Munich, Germany; 2 Biostatistics Group, Max Planck Institute of Psychiatry, Munich, Germany; Brunel University, United Kingdom

## Abstract

**Background:**

Tetratricopeptide repeat (TPR) motif containing co-chaperones of the chaperone Hsp90 are considered control modules that govern activity and specificity of this central folding platform. Steroid receptors are paradigm clients of Hsp90. The influence of some TPR proteins on selected receptors has been described, but a comprehensive analysis of the effects of TPR proteins on all steroid receptors has not been accomplished yet.

**Methodology and Principal Findings:**

We compared the influence of the TPR proteins FK506 binding proteins 51 and 52, protein phosphatase-5, C-terminus of Hsp70 interacting protein, cyclophillin 40, hepatitis-virus-B X-associated protein-2, and tetratricopeptide repeat protein-2 on all six steroid hormone receptors in a homogeneous mammalian cell system. To be able to assess each cofactor's effect on the transcriptional activity of on each steroid receptor we employed transient transfection in a reporter gene assay. In addition, we evaluated the interactions of the TPR proteins with the receptors and components of the Hsp90 chaperone heterocomplex by coimmunoprecipitation. In the functional assays, corticosteroid and progesterone receptors displayed the most sensitive and distinct reaction to the TPR proteins. Androgen receptor's activity was moderately impaired by most cofactors, whereas the Estrogen receptors' activity was impaired by most cofactors only to a minor degree. Second, interaction studies revealed that the strongly receptor-interacting co-chaperones were all among the inhibitory proteins. Intriguingly, the TPR-proteins also differentially co-precipitated the heterochaperone complex components Hsp90, Hsp70, and p23, pointing to differences in their modes of action.

**Conclusion and Significance:**

The results of this comprehensive study provide important insight into chaperoning of diverse client proteins via the combinatorial action of (co)-chaperones. The differential effects of the TPR proteins on steroid receptors bear on all physiological processes related to steroid hormone activity.

## Introduction

Steroid hormones are lipophilic signalling molecules, mediating a vast variety of physiological effects that depend on the cellular context of the target tissue. They act via steroid hormone receptors (SR), which belong to the nuclear receptor superfamily of ligand-activated transcription factors and serve as regulators of various target genes [1]–[3]. Upon binding to hormone, SR accumulate in the nucleus and either enhance or decrease transcription by interacting with their cognate DNA elements or by “cross-talk” with other transcription factors [4]–[6].

Hormone binding and activity of SR is shaped by molecular chaperones [7]. In general, molecular chaperones are highly conserved and abundant proteins that change the folding energy landscape for their client proteins to assist them in reaching their native conformation in an efficient and timely manner [8], [9]. SR interact with a heterocomplex consisting of the heat shock protein (Hsp) 90, Hsp70, Hsp40, Hsp70/Hsp90 organizing protein (HOP), p23 and various cochaperones in a stepwise fashion to attain a conformational state competent of binding to hormone with high affinity [10]. The model that emerged from research over the last two decades states that the initial folding steps are aided by Hsp70 based chaperones and co-chaperones, while the final steps are expedited through Hsp90-centred heterocomplexes [11].

Both Hsp70 and Hsp90 feature a C-terminal EEVD motif that serves as acceptor site for cochaperones that harbour a tetratricopeptide repeat (TPR) domain [12]. In particular the Hsp90-interacting TPR proteins have received broad attention as proposed regulators of SR function [11], [13]. Among these TPR proteins are the carboxyl terminus of Hsc70-interacting protein (CHIP), Cyclophillin-40 (Cyp40), the immunophilin FK506-binding proteins (FKBP) 51 and 52, protein phosphatase 5 (PP5), the tetratricopeptide repeat protein 2 (TPR2) and the hepatitis virus B X-associated protein 2 (XAP2).

Many of the TPR proteins bring additional molecular functions to the SR-chaperone heterocomplexes. CHIP contains a C-terminal U-box that interacts with ubiquitin-conjugating enzymes and has been reported to promote degradation of various steroid receptors [14]–[17]. The immunophilin and peptidylprolyl isomerase (PPIase) Cyp40 has also been identified in SR-heterocomplexes, but its role regarding SR-function is still unclear [18]. FKBP51, another PPIase, was characterised as a cellular factor contributing to the glucocorticoid resistance observed in New World primates [19], [20]. It inhibits glucocorticoid receptor (GR) activity by lowering hormone binding affinity of the receptor and delaying nuclear translocation [20], [21], and also inhibits the mineralocorticoid receptor [22]. In contrast, the highly homologous FKBP52 was found to positively modulate SR function and to be critical for progesterone and androgen function *in vivo* [18], [23]–[27]. While the PPIase protein domains of FKBP51 and FKBP52 play an important role in GR's regulation, the function of the enzymatic PPIase activity remains enigmatic [18], [21], [28].

PP5 is the only TPR-domain containing phosphatase identified so far; it has been shown to modulate a variety of cellular pathways [29]. The role of PP5 in SR signalling appears controversial so far, possibly due to the different approaches and experimental systems. Both positive and negative modulatory effects of PP5 on steroid dependent transcription have been reported. Down-regulation of PP5 expression was shown to increase GR activity in reporter gene assays [30] and transcription of estrogen receptor (ER) target genes [31] suggesting a negative modulatory role of PP5 in steroid dependent signalling. In contrast, in a different study siRNA-mediated PP5 knock-down lead to a decrease in transcription of GR target genes [32]. In yeast, the PP5 homolog Ppt1 acts as a positive modulator of GR, possibly by removing inhibitory phosphates from Hsp90 [33].

TPR2 is a J-domain containing cochaperone which has been demonstrated to modulate GR and PR signalling [34], [35]. It may act by mediating the retrograde transfer of substrates from Hsp90 onto Hsp70 [34]. XAP2 has been well studied for its role in regulating the activity of the arylhydrocarbon receptor (AhR) class of nuclear receptors [36]. Recently, XAP2 was shown to inhibit GR-mediated transcription [37].

Based on evidence from the literature and our own studies on the GR-inhibitory role of FKBP51, we had initiated a genotyping study that revealed a genetic association of this TPR protein with the response to medication in major depression [38]. Meanwhile, FKBP51 has been included in several studies and attracted great attention for the association of its genetic polymorphisms and gene expression level with a number of stress-related phenotypes and neuropsychological diseases, such as major depression or post traumatic stress disorder [38]–[46]. All these findings corroborate a physiological role of FKBP51 in stress regulation, most likely via its action in GR signalling.

Since several TPR proteins should be able to compete with FKBP51 for binding to the binding site for TPR proteins in an Hsp90 dimer [47]–[49], we assume that the overall impact of FKBP51, or any other TPR protein, on GR, or SR in general, depends on the relative abundance and mode of action of the other TPR proteins present in the same cell. Knowledge about each of these factors' capability to influence SR function might provide the basis for the understanding of tissue responsiveness to steroid hormones. A comprehensive comparison of the TPR-proteins on the function of all steroid receptors in a homogenous mammalian system has not been accomplished yet. Thus, we assessed the impact of the TPR proteins CHIP, Cyp40, FKBP51, FKBP52, PP5, TPR2 and XAP on each of the SR.

## Results

### Different responsiveness of the steroid hormone receptors in reporter gene assay

To set up an assay for the determination of the influence of the seven selected TPR proteins CHIP, CYP40, FKBP51, FKBP52, PP5, TPR2 and XAP2 on the six steroid receptors GR, MR, PR, AR, ERα and ERβ, we established reporter gene assays for each of the receptors. For GR, MR, PR and AR, we made use of the hormone-responsive elements of the MMTV LTR promoter that was linked to the structural part of the firefly luciferase gene [50]. To measure the activity of the two ER receptors we used a luciferase reporter plasmid with two copies of an estrogen responsive element instead of the MMTV LTR [51]. For each receptor, we used two sub-saturating concentrations of hormone as well as one concentration well in the range of saturation.

**Figure 1:**
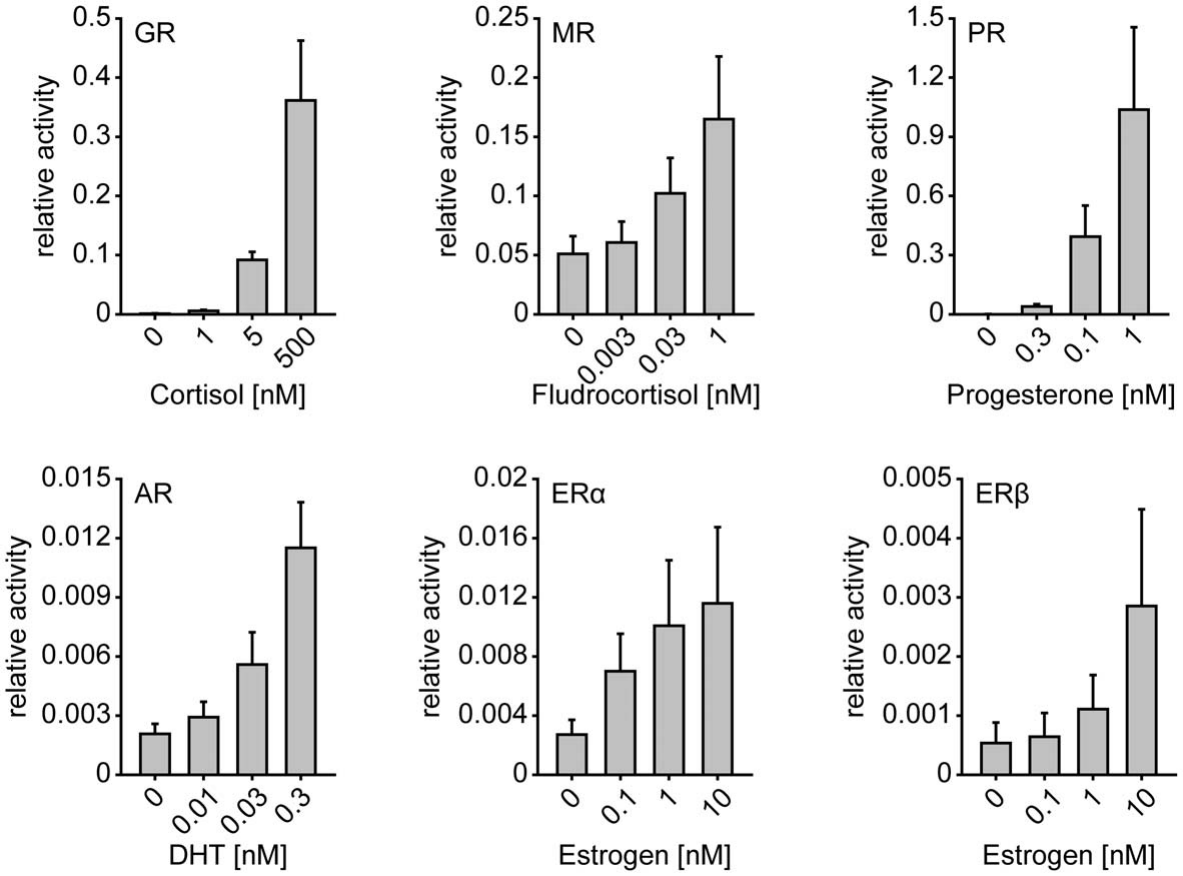
Response of steroid hormone receptors in MMTV-reporter gene assays. Neuronal SK-N-MC cells were transfected with a plasmid expressing one of the HA-tagged SRs, the MMTV firefly-luciferase reporter plasmid when transfecting GR, MR, PR, or AR, an ERE firefly-luciferase reporter plasmid for ERα and ERβ, and the Gaussia-KDEL control plasmid. After transfection, the cells were cultivated for 24 h in the presence of the indicated concentrations of hormone (DHT: Dihydrotestosterone) or EtOH as solvent control. Receptor activity represents firefly data normalized to Gaussia activities + S.E.M. of at least four independent experiments, each performed in duplicate.

GR and PR displayed the widest, AR a considerable, and MR and the two ERs a moderate range of hormone inducible activity in human neuronal SK-N-MC cells (Fig. 1). We chose this cell line for two reasons, first because is largely devoid of steroid receptors, and second because of its neuronal origin.

Since the effects of FKBP51 on GR have been reported to be most pronounced at sub-saturating concentrations of hormone, we focused our further analyses on conditions that yielded significant, but not yet full activation of the respective steroid receptor. In addition, we also included one saturating concentration of hormone for each receptor.

### Steroid receptors display differential sensitivity to TPR-proteins

**Figure 2:**
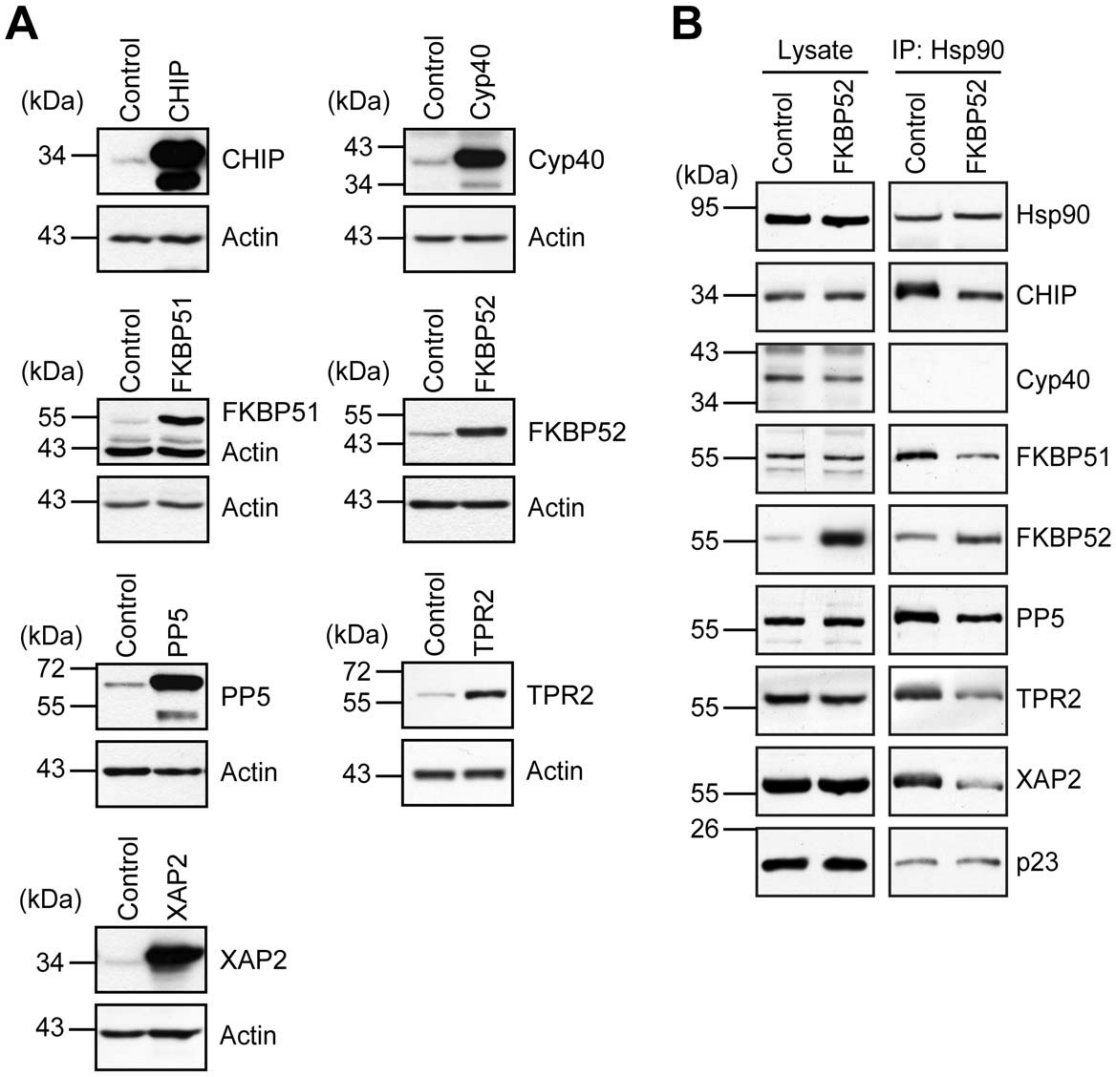
TPR proteins are significantly enhanced upon ectopic expression and change Hsp90 heterocomplex composition. A, SK-N-MC cells were transfected with plasmid expressing one of the TPR proteins, lysed after 48 h and levels of the respective TPR protein was determined by Western blot analysis. B, HEK-293 cells were transfected with FLAG tagged Hsp90 along with FKBP52 expressing plasmid or control plasmid. Hsp90 was precipitated from lysates and the levels of co-precipitated cofactors were determined by Western blot.

To assess the effect of the TPR proteins on steroid receptor activity, each of the FLAG-tagged TPR proteins was co-expressed with each of the HA-tagged steroid receptors GR, MR, PR, AR, ERα, or ERβ, respectively, along with reporter and control plasmids. Since mammalian cells, in contrast to yeast, feature a number of different receptor-relevant TPR proteins, we reasoned that overexpression of a specific TPR protein is necessary to significantly enhance occupancy of the TPR acceptor site on Hsp90 by this specific cofactor. To test whether this is indeed the case under the conditions chosen we first evaluated the degree of overexpression for each of the TPR cofactors (Fig. 2A). Cells were transfected with plasmids encoding for one of the TPR proteins and probed their abundance in cell lysates using Western blot analysis. Each of the TPR cofactors was at least 4 fold enhanced over the endogenous levels (Fig. 2A).

Since our experimental design was further based on the assumption that selectively enhancing the level of one of the TPR cofactor results in changing the composition of the Hsp90 heterocomplexes, we tested this at the example of FKBP52 overexpression. Cells were transfected with FKBP52 expressing plasmid, and Hsp90 complexes were immunoprecipitated from cell lysates of FKBP52 overexpressing cells and control cells. While more FKBP52 was co-precipitated with Hsp90 complexes, all the other investigated TPR cofactors were less abundant (Fig. 2B, Cyp40 was below detection limit). As an important control, the interaction of Hsp90 with the non TPR protein p23 was not changed by increasing FKBP52 (Fig. 2B).

**Figure 3:**
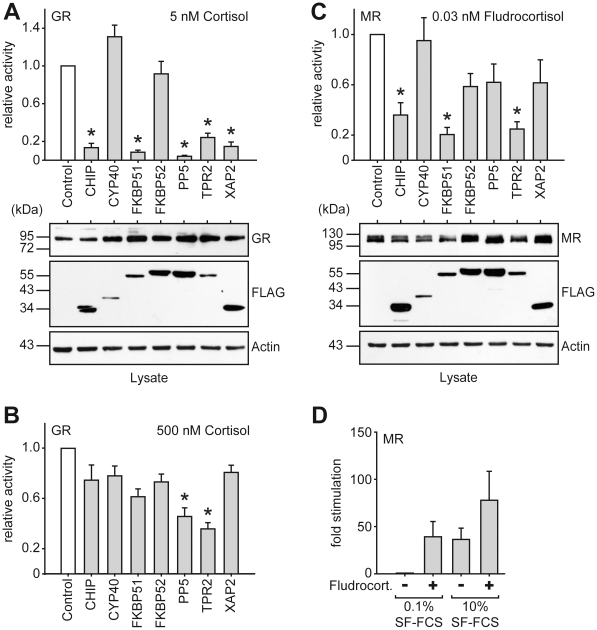
GR and MR activities in the presence of different TPR-proteins. A-C, SK-N-MC cells in 96 well plates were transfected with the MMTV-Luc, Gaussia-KDEL control plasmid, a plasmid expressing one of the HA-tagged steroid hormone receptor (GR in A and B, MR in C and D) and constant amounts (200 ng) of a plasmid expressing one of the FLAG-tagged TPR-proteins. After transfection, the cells were cultivated for 24 h in the presence of hormone or vehicle as indicated. Relative receptor activity represents firefly data normalized to Gaussia activities and presented as relative stimulation to control + S.E.M. of at least four independent experiments performed in duplicate. Control cells were transfected with cloning plasmid instead of the TPR protein expressing plasmid. Lower panels of A and C, immunoblot of cell extracts, probed with anti-HA antibody visualizing steroid receptor expression, the same membrane probed with FLAG antibody demonstrating expression of TPR proteins and with actin antibody as loading control. D, After transfection, cells were cultivated in 0.1% or 10% SF-FCS containing media for 24 h in the presence of 0.03 nM fludrocortisol, or EtOH as vehicle control. Firefly luciferase data were normalized to Gaussia luciferase activities and are presented as relative stimulation + S.E.M. of three independent experiments performed in triplicate. * denotes *p-*values ≤0.001.

**Figure 4:**
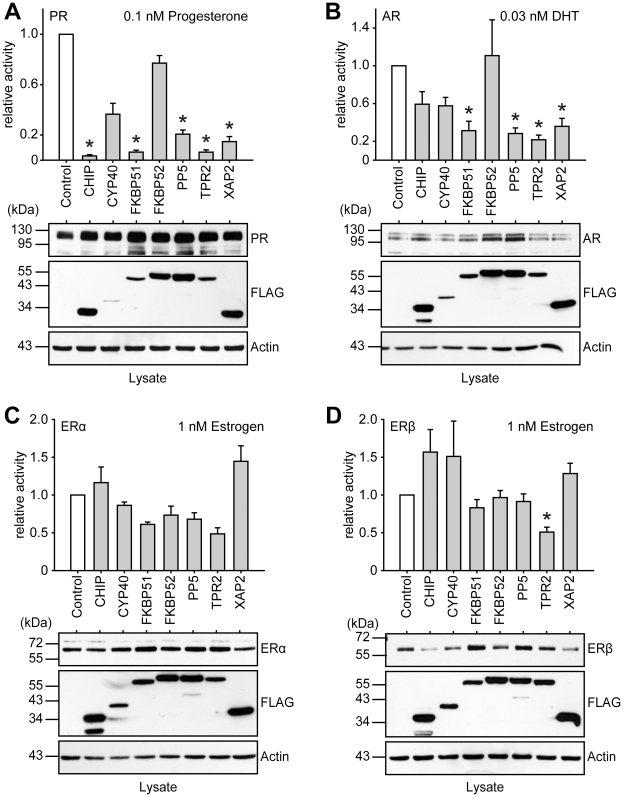
PR, AR, ERα and ERβ activities in the presence of different TPR proteins. SK-N-MC cells were transfected with the MMTV-Luc (for PR and AR assays), or the ERE-Luc reporter plasmid (for ERα and ERβ assays), the Gaussia-KDEL control plasmid, a plasmid expressing the HA-tagged steroid hormone receptor as indicated and the plasmid expressing a FLAG-tagged TPR-protein. After transfection, cells were cultivated for 24 h in the presence of hormone as indicated. Relative receptor activity represents firefly data normalized to Gaussia activities and presented as relative stimulation to control + S.E.M. of at least four independent experiments performed in duplicate. Control cells were transfected with cloning plasmid replacing the TPR protein expression plasmid in the transfection mixture. Lower panels of A–D display immunoblots of cell extracts, probed with anti-HA antibody visualizing steroid receptor expression, the same membrane probed with FLAG antibody demonstrating expression of the TPR proteins and with actin antibody as loading control. * denotes *p*-values ≤0.001.

The first observation we made in the reporter gene assays was that, in general, the changes in receptor activity upon co-expression of TPR cofactors were more pronounced for GR, MR, and PR than for AR, and even more than for the two ERs, which were almost not affected (Figs. 3 and 4). Strong inhibitors of GR were CHIP, FKBP51, PP5, TPR2, and XAP2, while CYP40 and FKBP52 showed virtually no effect (Fig. 3A). As shown for GR, at saturating concentrations of hormone the inhibitory effect was greatly diminished, even though TPR2, for example, still reduced GR's activity twofold (Fig. 3B). Similar observations were made with saturating concentrations of hormone at the other receptors (data not shown).

The TPR reactivity profile of MR was very similar, except for PP5 and XAP, which exerted only a marginally inhibitory effect on MR (Fig. 3C). Albeit we were using stripped serum free of steroids, we observed a significant activity of MR even in the absence of added hormone, which was affected by the TPR cofactors in the same way as the hormone-stimulated activity (Fig. 3C and data not shown). To test whether any serum component might have contributed to hormone-independent activation of MR we cultivated transfected cells in serum free media for 24 h before measuring reporter activity. Serum withdrawal reduced MR-dependent transactivation, suggesting that factors other than glucocorticoids are present in steroid free media which partially activate MR's transcriptional activity (Fig. 3D). This effect appeared to be additive to the glucocorticoid-mediated effect, because stimulation with sub-saturating concentrations of fludrocortisol (0.03 nM) in stripped serum containing media resulted in higher MR activation than in serum free media (Fig. 3D).

Similarly to GR, PR showed the highest activity when co-expressed together with CYP40 or FKBP52 (Fig. 4A). The effects of the TPR-cofactors were noticeably attenuated in the case of AR (Fig. 4B). Only co-expression of FKBP52 maintained AR activity, while all the other TPR cofactors reduced this receptor's activity to a moderate degree, with TPR2 being the strongest inhibitor (5 fold inhibition, Figure 4B). ERα and ERβ showed almost no reaction to the presence of TPR cofactors under our conditions, except for TPR2, which reduced the activity of these receptors about 2 fold (Figs. 4C, D).

We also monitored the expression levels of the co-expressed receptors and TPR proteins. There were some variations throughout the experiments, but overall there were no gross alterations in the levels of the steroid receptors in dependence of the co-expressed TPR cofactor, except for CHIP which often, albeit not consistently, led to lower receptor expression levels (Figs. 3 and 4 examples in the panels below the activity assay graphs). This was not unexpected, because CHIP has been identified as E3 ligase and been shown to reduce the levels of GR [15]. The levels of the co-expressed TPR proteins also varied between experiments. Overall, Cyp40 and TPR2 had a tendency to be expressed at lower levels, and to a lesser extent also FKBP51, while the other cofactors expressed at the same levels.

### The estrogen receptors show little sensitivity to geldanamycin

**Figure 5:**
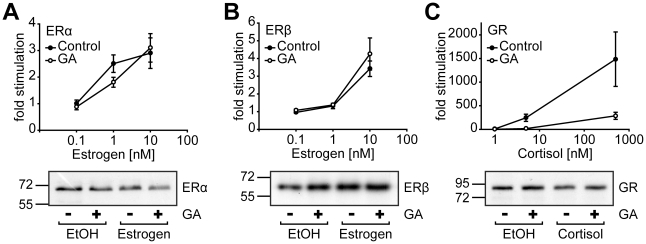
Estrogen receptors display little sensitivity to the Hsp90 inhibitor geldanamycin. SK-N-MC cells were transfected with 0.25 µg of one of the plasmids expressing ERα (A), ERβ (B) or GR (C), together with either ERE-Luc (A,B) or MMTV-Luc (C) as reporter plasmid and the Gaussia-KDEL control plasmid. After transfection, the cells were cultivated for 24 h in the presence of hormone and 10 ng/ml GA as indicated. Relative receptor activity represents Firefly data normalized to Gaussia activities and is presented as relative stimulation to control + S.E.M. of at least four independent experiments performed in duplicate. Lower panels, analysis of receptor expression after GA treatment in the presence or absence of hormone (10 nM estrogen, 500 nM cortisol).

Since most of the TPR proteins had little impact on ERs' transcriptional activity, we wondered whether these two receptors are dependent on functional Hsp90 at all under our assay conditions. Therefore, we applied the specific Hsp90 inhibitor geldanamycin (GA), which has been shown to block Hsp90 activity by binding to its ATP pocket [52], [53]. We performed reporter gene assays for ERα, ERβ, and GR in the presence or absence of GA (Fig. 5). While GA efficiently reduced the activity of GR (Fig. 4C), it had very little effect, if any, on the activity of ERα and ERβ (Figs. 5A,B). Whatever the reason for the apparent Hsp90-independent action of these two receptors is, it could explain why the Hsp90 cofactors have so little impact on their activity.

It has been found that GA leads to degradation of Hsp90 client proteins such as GR [54]–[56]. To test whether the differential responsiveness of GR and ER to GA is also reflected on the level of protein stability, we measured the expression levels of GR and ER upon GA treatment. Neither GR nor ER were significantly changed in their protein levels after exposure of transfected HEK cells (Fig. 5) or SK-N-MC cells (data not shown) with GA, which could be due to the much lower concentrations of GA used here compared to other studies [54], [55].

### Cyclophilin 40 is unable to rescue receptor activity

**Figure 6:**
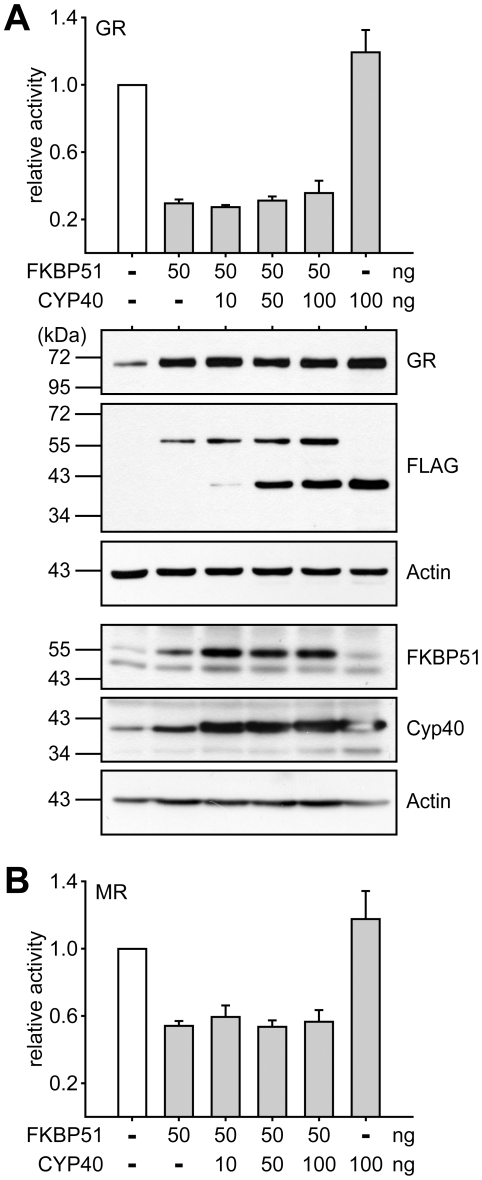
Cyp 40 is unable to compete the inhibitory effect of FKBP51. SK-N-MC cells were transfected with the MMTV-Luc reporter plasmid, the Gaussia-KDEL control plasmid, one of the plasmids expressing the HA-tagged GR or MR as indicated, and plasmids expressing FKBP51 and Cyp40 at the indicated amounts. After transfection, the cells were cultivated for 24 h in the presence of 10 nM cortisol (A) or 0.03 nM Fludrocortisol (B). Bar graphs indicate the relative reporter activity representing Firefly measurements normalized to Gaussia activities and presented as relative stimulation + S.E.M. of three independent experiments performed in triplicate. Lower panel of A displays immunoblots of cell extracts, probed with HA antibody demonstrating GR expression and the same membrane probed with FLAG antibody to detect overexpressed FKBP51 and Cyp40, and actin as control. In addition, antibodies directed against FKBP51 or Cyp40 were used to visualize the combined levels of endogenous and ectopic TPR protein.

FKBP52, which does not change the activity of GR when co-expressed with this receptor in mammalian cells (Fig. 3A, and [21]), has been shown to be able to attenuate the inhibitory effect of FKBP51 [21]. In our screen of the activity profiles of the TPR proteins Cyp40, like FKBP52, had no or very little effect on steroid receptors (Figs. 3 and 4). Thus, the question arose, whether an effect of Cyp40 on GR or MR may become apparent under conditions of compromised receptor activity, i.e. when an inhibitory protein is co-expressed. Therefore, we coexpressed FKBP51 at moderate levels to inhibit GR and MR, and added increasing amounts of Cyp40 expressing plasmid (Fig. 6). Even though Cyp40 was expressed up to levels that exceeded those of FKBP51 (both proteins were FLAG tagged, allowing a direct comparison in a Western blot) and well above endogenous levels, it was unable to rescue the activity of GR or MR (Figs. 6A and B). This is in contrast to FKBP52, which has been shown to be able to revert the inhibitory action of FKBP51 [21].

### Binding profiles of TPR proteins to steroid receptor-Hsp90 heterocomplexes

The ability of TPR proteins to access heterocomplexes of steroid receptors and Hsp90 is assumed as prerequisite for their impact on these receptors. Therefore, we evaluated the relative incorporation of the TPR-proteins into steroid receptor complexes employing complementary co-immunoprecipitation. The estrogen receptors were not included, because they were only marginally affected by most of the TPR proteins. We expressed each of the HA-tagged steroid receptors in combination with each of the seven FLAG-tagged TPR proteins and performed co-immunoprecipitations with antibodies directed against the HA-tagged receptors or the FLAG-tagged TPR proteins, respectively, and visualized co-precipitated proteins by Westernblot analysis.

Since we observed varying efficiencies in the amount of precipitated protein using HA- or FLAG-directed antibodies, co-precipitated proteins were normalized to the precipitated primary target, and in case of HA-directed IPs also to the relative expression of the different TPR proteins.

**Figure 7 pone-0011717-g007:**
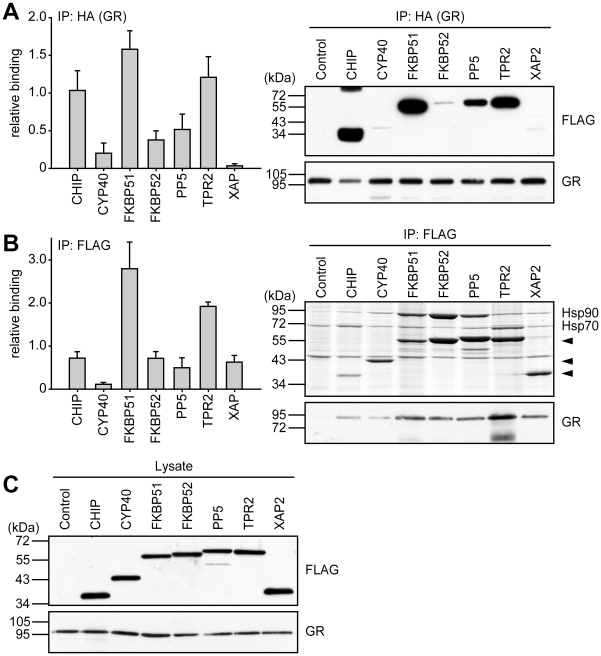
TPR-proteins differently interact with GR heterocomplexes. HEK-293 cells were transfected with 5 µg of a plasmid expressing HA-tagged GR together with 2-10 µg (to achieve similar expression levels) of one of the plasmids expressing a FLAG-tagged TPR protein. After 48-72 h cultivation in SF-FCS containing media, cells were harvested, lysed, and protein extracts prepared for immunoprecipitation of either the HA-tagged GR (A), or the FLAG-tagged TPR-proteins (B). A, Precipitation of HA-GR. Displayed is an example of an immunoblot that was probed with FLAG antibody to visualize co-precipitated TPR-proteins (upper right panel), and an immunoblot of the same membrane probed with HA antibody demonstrating precipitated GR (lower right panel). Left panel, quantification of the relative binding of the TPR-proteins to the steroid receptor heterocomplexes. FLAG- and HA-immunoblot signals of the eluates and FLAG immunoblot signals of the cell extracts, demonstrating expression of TPR proteins (C), were scanned and subjected to densitometry. The signal from the co-precipitated FLAG protein was corrected first by the amount of precipitated receptor and second by the amount of the TPR-protein present in the respective cell extract. Binding of TPR-proteins is presented relative to the mean of the normalized FLAG-eluate signals of CHIP, FKBP51, FKBP52, and PP5. Quantification represents the means of three independent experiments +S.E.M. B, precipitation of TPR proteins. Upper right panel, coomassie stained gel of eluates visualizing precipitated TPR-proteins (arrowheads) and co-precipitated Hsp90 and Hsp70. Lower right panel, immunoblots of eluates probed with HA antibody to demonstrate binding of GR to TPR-protein heterocomplexes. Left panel, quantification of the relative binding of co-precipitated proteins to the precipitated TPR-proteins. For quantification, signals were scanned and subjected to densitometry. Each HA immunoblot signal of the eluate was corrected by the amount of precipitated TPR-protein. Binding of steroid receptors is presented relative to the mean of the corrected HA eluate signals. Quantifications represent means of three independent experiments +S.E.M.

For GR, the receptor IP revealed CHIP, FKBP51 and TPR2 as strong binders to the heterocomplex (Fig. 7). The FLAG-IPs targeting the TPR proteins revealed a similar binding pattern. We observed that Cyp40 exhibited weak interaction with the heterocomplex, which may account for its inability to compete the inhibitory effect of FKBP51. Notably, the strongest binders all were strongly inhibitory proteins. On the other hand, PP5, which also significantly reduced GR activity, in comparison displayed only moderate interaction.

**Figure 8 pone-0011717-g008:**
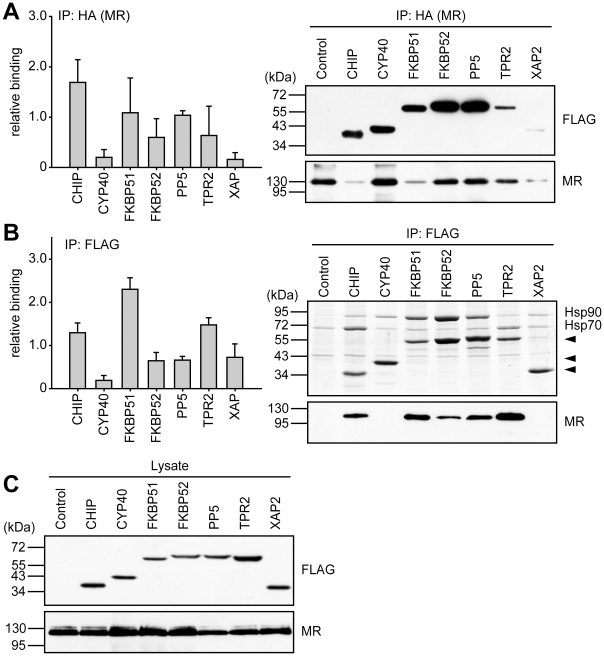
Differential interaction of TPR-proteins with MR heterocomplexes. HEK-293 cells were transfected as described for figure 7, except that HA-MR was expressed instead of HA-GR. Cells were processed and protein interactions were analyzed also as described for figure 7. In A, binding of TPR-proteins is presented relative to the mean of the normalized FLAG-eluate signals of CHIP, FKBP51, FKBP52 and PP5. Quantification represents means of three independent experiments (two for TPR2) +S.E.M.. In B, binding is normalized as in figure 7. C, FLAG- and HA-immunoblot signals of the cell extracts, demonstrating expression of TPR proteins and MR. Quantifications represent means of three independent experiments +S.E.M.

For MR, the interaction pattern of the TPR cofactors was similar to that of GR. Again, CHIP, FKBP51 and TPR2 exhibited strong interaction, while Cyp40 showed very little binding, both when immunoprecipitating the receptor or the cofactor (Fig. 8 A and B). Of note, the inability of PP5 to inhibit MR's transcriptional activity was not reflected by a corresponding low incorporation into MR heterocomplexes.

**Figure 9 pone-0011717-g009:**
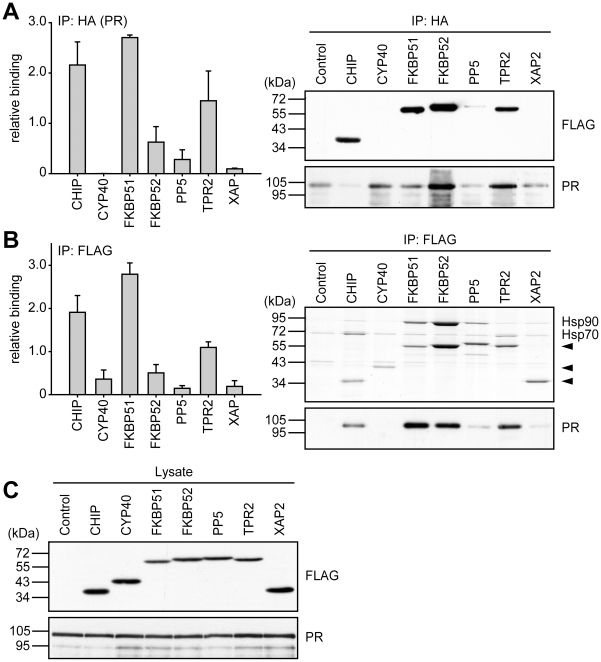
Differential interaction of TPR-proteins with PR heterocomplexes. HEK-293 cells were transfected as described for figure 7, except that HA-MR was expressed instead of HA-GR. Cells were processed and protein interactions were analyzed also as described for figure 7. In A, binding of TPR-proteins is presented relative to the mean of the normalized FLAG-eluate signals of CHIP, FKBP52, PP5 and TPR2. Quantification represents means of three independent experiments (two for FKBP51) +S.E.M. In B, binding is normalized as in figure 7. C, FLAG- and HA-immunoblot signals of the cell extracts, demonstrating expression of TPR proteins and PR. Quantifications represent means of three independent experiments +S.E.M.

In the case of PR, we observed the strongest interaction with the PR-Hsp90 heterocomplex for CHIP, FKBP51, and TPR2 (Fig. 9), which in the reporter gene assay also were the ones that exhibited the strongest inhibitory activity (Fig. 4A). Remarkably, XAP2 and PP5, which also reduced PR's transcriptional activity, bound only weakly to the complex.

**Figure 10:**
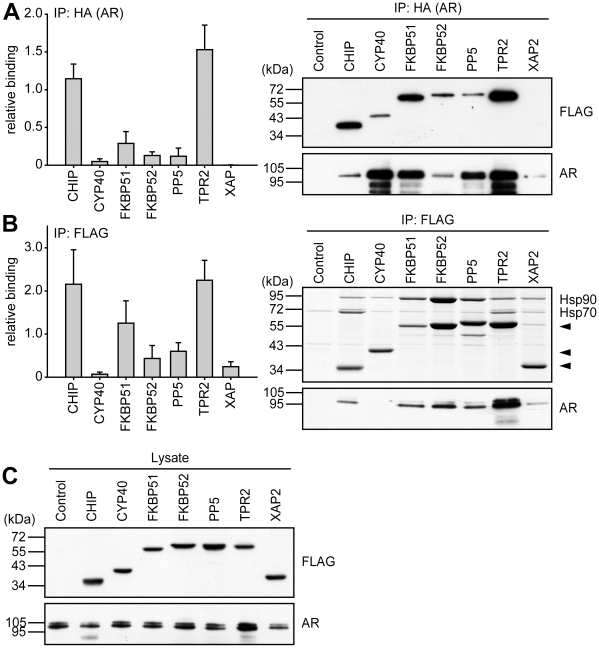
Differential interaction of TPR-proteins with AR heterocomplexes. HEK-293 cells were transfected as described for figure 7, except that HA-MR was expressed instead of HA-GR. Cells were processed and protein interactions were analyzed also as described for figure 7. In A, binding of TPR-proteins is presented relative to the mean of the normalized FLAG-eluate signals of CHIP, FKBP51, and TPR2. Quantification represents means of three independent experiments (two for XAP2) +S.E.M. In B, binding is normalized as in figure 7. C, FLAG- and HA-immunoblot signals of the cell extracts, demonstrating expression of TPR proteins and AR. Quantifications represent means of three independent experiments +S.E.M.

Although AR showed less activity change in response to co-expression of TPR cofactors than GR, MR and PR, the TPR cofactors exhibited a distinct binding profile (Fig. 10). The most efficient binding was observed for TPR2, in the presence of which AR was least transcriptionally active (Fig. 4B). In general though, there was no strict correlation between binding efficiency to the Hsp90-AR heterocomplex and the influence on the transcriptional activity of AR. For example, in the presence of CHIP or Cyp40, AR exhibited a very similar transcriptional activity, but these two TPR proteins differed markedly in their ability to access the AR-Hsp90 heterocomplex (Figs. 4B and 10).

### TPR cofactors favor differently composed multi-chaperone heterocomplexes

During maturation, the steroid receptor proceeds through a multi-chaperone machinery in which each step is characterized by a relative abundance of distinct chaperones [7]. Therefore, it is possible that preference of the TPR cofactors to distinct heterochaperone complex compositions represents an important mechanistic aspect of their function. Thus, we compared the abundance of endogenous components of the chaperone machinery co-precipitating with the immunoadsorbed TPR-cofactors.

Since the FLAG-tagged proteins were precipitated with different efficiencies (although amounts of plasmids were adjusted so that the TPR proteins were expressed at similar levels, compare Figs. 7–10), the amount of co-precipitated Hsp90, Hsp70 and p23 was normalized to the amount of precipitated TPR cofactor. We consistently observed some nonspecific binding of Hsp70 to the FLAG agarose resin, and therefore, considered only levels exceeding the background binding as indicative of Hsp70 interaction. Co-expression of the different steroid receptors did not change the relative co-precipitation of Hsp70, Hsp90 and p23. Therefore, we used the results of experiments with different steroid receptors to determine the relative binding of these components to the TPR-proteins.

**Figure 11:**
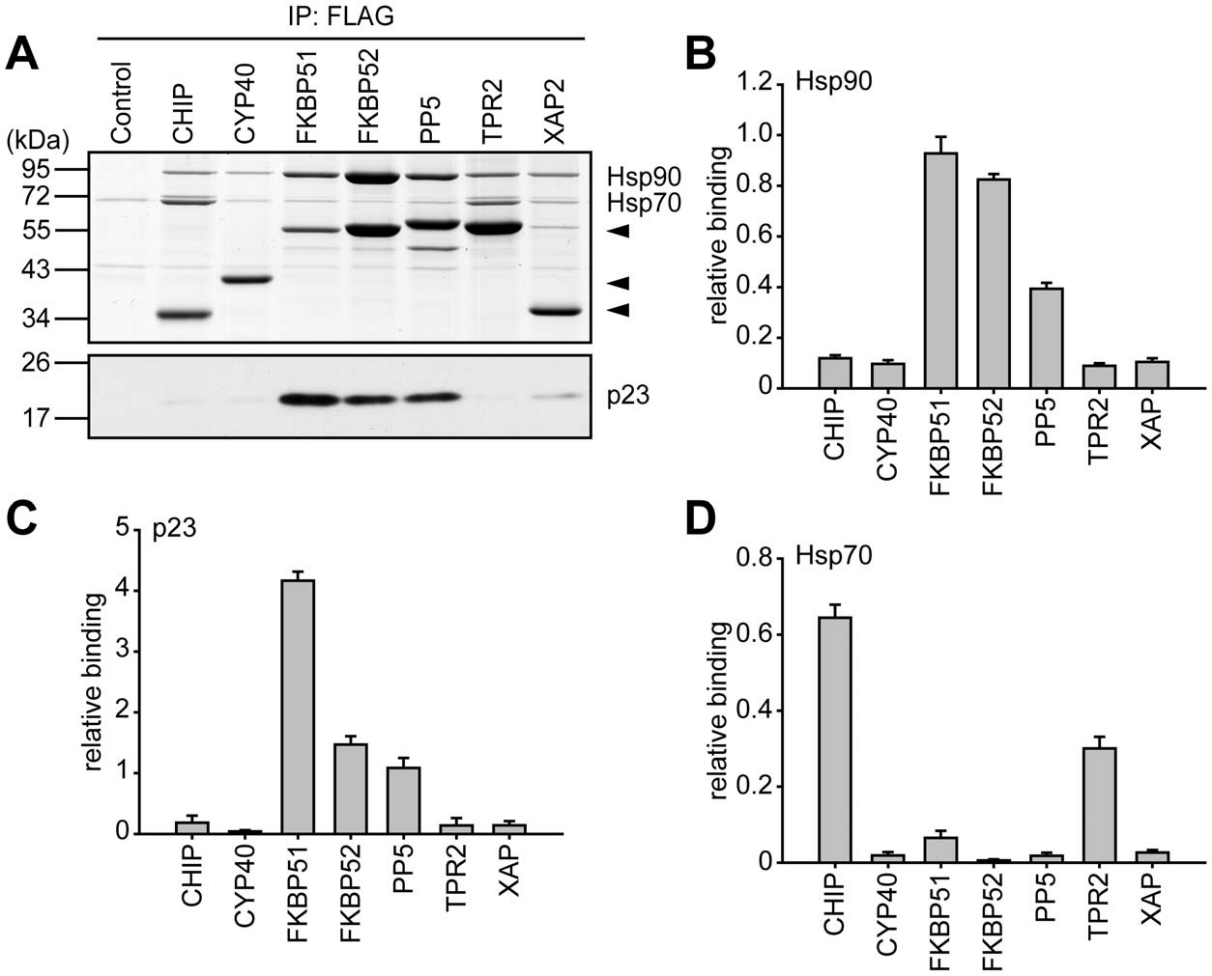
TPR cofactors differentially recruit components of the multichaperone heterocomplex. HEK cells were transfected and TPR cofactors immunoprecipitated as described in the legends to figures 7–10. The relative amounts of the precipitated TPR cofactors, the co-precipitated Hsp70 and Hsp90 were determined by densitometry of a coomassie stained gel of the eluates (A, upper panel), and the relative amount of p23 by densitometry of the immunoblot signals (A, lower panel). B and D, quantification of the relative binding of co-precipitated Hsp90 (B) and Hsp70 (D). Hsp90 signals and Hsp70 signals (only intensities above background binding were taken into consideration) were normalized to signals of the respective precipitated TPR cofactors. Data are presented as relative binding + S.E.M. of at least 12 independent experiments with different steroid receptors. C, quantification of the relative binding of co-precipitated p23. The p23 immunoblot signals were related (normalized) to the respective TPR cofactor signal. Binding of p23 is presented relative to the mean of the normalized p23 eluate signals of the complete set of TPR-proteins.

Hsp90 interaction was detected for all TPR cofactors investigated here, as expected. However, there was a considerable difference in the relative amount of co-precipitated Hsp90 (Fig. 11A and B). FKBP51 and FKBP52 displayed the strongest Hsp90 interaction, and PP5 still about 4 fold higher interaction than CHIP, CYP40, TPR2 and XAP2, which all bound at comparable levels. Notably, while p23 interaction reflected the relative Hsp90 co-precipitation in general, p23 co-precipitated with FKBP52 less than with FKBP51 or PP5 in relation to the Hsp90 association (Fig. 11C). Apparently, FKBP51 and PP5 favor p23 containing Hsp90 heterocomplexes more than FKBP52, possibly by stabilizing the interaction between Hsp90 and p23.

Hsp70 binding was detected for CHIP and TPR2, as reported previously [34], [57]. No Hsp70 binding was detected for CYP40, FKBP52, PP5 and XAP2, but surprisingly, for FKBP51. Although this binding was clearly weaker than that observed for CHIP and TPR2, it was significantly more than the virtually non existent Hsp70 binding of FKBP52 (p = 0.007 in an unpaired student's t-test). This could indicate that FKBP51 favors early stages of the folding cycle, which could contribute to its inhibitory function.

### Loss of FKBP52 impairs GR function

**Figure 12:**
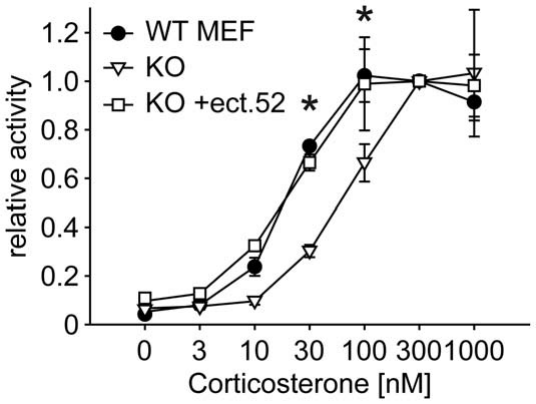
Loss of FKBP52 affects GR responsiveness to cortisol. FKBP52-KO MEF cells (open symbols) or WT MEF cells (closed circles) were transfected with the MMTV-Luc reporter plasmid, the Gaussia-KDEL control plasmid, a plasmid expressing the HA-tagged mGR and either a plasmid expressing FLAG-tagged FKBP52 (+ect.52) or empty vector. After transfection, cells were cultivated for 24 h in the presence of hormone. Relative receptor activity represents firefly data normalized to Gaussia activities and is presented relative to the activity at saturating 300 nM corticosterone +S.E.M. of three independent experiments, each performed in triplicates. Significance of different receptor activation between FKBP52 KO cells and FKBP52 KO cells ectopically expressing FLAG-tagged FKBP52 was evaluated by one sampled T-test (* denotes *p*-values ≤0.001).

The experiments described so far were based on increasing the abundance of a specific TPR cofactor in Hsp90 heterocomplexes. Considering the plethora of TPR cofactors in the cell, we pondered on the ability of mammalian cells to compensate for the loss of one of the proteins. Based on the inability of enhanced FKBP52 to significantly increase GR function, we reasoned that loss of an inhibitory factor, for example FKBP51, would have little effect. Therefore, we experimentally addressed the effect of loss of the established positive GR regulator FKBP52. Since our attempts to reduce FKBP52 using si-RNA resulted in only partial reduction (data not shown), we used mouse embryonic fibroblast (MEF) FKBP52 KO and WT cells. We found that stimulation of GR activity at saturating concentrations of hormone was not significantly affected (data not shown). However, higher concentrations of hormone were needed in FKBP52 ko cells to elicit a GR response comparable to that in WT MEF cells (Fig. 12).

Since cells derived from different animals and cultivated for several generations can differ in numerous factors, it was mandatory to test whether the difference in the cortisol responsiveness between WT and FKBP52 KO MEF cells was indeed due to loss of FKBP52. Therefore, we overexpressed FKBP52 in FKBP52 KO MEF cells, which rendered the cortisol responsiveness indistinguishable from that of WT MEF cells (Fig. 12).

## Discussion

How are molecular chaperones able to assist correct folding of a plethora of structurally divergent proteins? In general, the various chaperone factors protect non-native protein chains from misfolding and aggregation, but do not contribute conformational information to the folding process [8]. They interact with features of non-native protein folds that are common to many proteins, such as hydrophobic stretches and unstructured backbone regions, and provide nano-compartments to shield proteins during their folding process from other proteins. Hsp90 regulates mainly a wide range of signal transduction molecules, and thus belongs to the more specialised, but still very versatile chaperones [9], [58]. Our study provides a better understanding of this versatility through combinatorial compositions of the Hsp90-client heterocomplexes.

Of the six steroid receptors, the closely homologous GR, MR and PR exhibited the strongest reaction to changes in the TPR-protein make-up of the cell (Fig. 2 and 3). AR, and the ERs in particular, were less affected by co-expressing any of the co-chaperones. This may be explained by a diminished Hsp90-dependency of ER, at least in our cellular set-up, corroborated by the ineffectiveness of GA towards ER. Others have also provided evidence that ER may operate independently of Hsp90 [59], [60], which contrasts reports on lower ER activity where Hsp90 function was compromised [61]–[64]. It should be noted, though, that high doses of GA of 0.2–1 µg/ml have been used in these reports. We used a 20–100 fold lower concentration of GA, which efficiently reduced GR activity, like we also have observed previously [65]. We cannot exclude the possibility that ER activity could be impaired also in our cellular system at very high concentrations of GA, which however, would raise the question of non-specific effects of GA. We propose that the Hsp90-dependency of ER is cell-type dependent, and possibly affected by the presence or absence of additional, yet to be revealed factors. In addition, high doses of GA have been reported to induce reactive oxygen species in cells [66]–[69], which might contribute to differences in the effects of GA on ER at different concentrations.

Our study also documents numerous differences in the efficacies of the TPR proteins' influence on SR. Cyp40 exhibited only a minor effect on AR and PR, and no effect on GR, MR and the ERs, which concurs with its small binding affinity to Hsp90 and steroid receptor heterocomplexes in comparison to other TPR proteins (Fig. 6–10). Work in yeast, which expresses the two Cyp40 homologues Cpr6 and Cpr7, revealed an involvement of Cpr7, but not Cpr6 in the hormone-dependent activity of GR [70], [71]. In addition, Cpr6 did not influence Hsp90 activity [72]. In mammalian cells, the effect of Cyp40 on steroid receptors has not been directly assessed. However, cyclosporine A, which is known to target Cyp40 as well as Cyp18, somewhat diminished AR function in LNCaP cells [73].

CHIP efficiently inhibited the transactivational activity of GR, MR, PR, and moderately affected AR. It has been reported that CHIP induces degradation of GR, AR and ERα [15]–[17] and reduces hormone binding of GR [15]. With respect to steroid receptor degradation, we observed a tendency towards lower receptor amounts, but no consistently significant effect. Since saturating concentrations of hormone greatly attenuated the inhibitory effect of CHIP on all steroid receptors (Fig. 2B for GR and data not shown for MR, PR, AR), mechanisms in addition to protein degradation must be responsible for the observed inhibition, most likely reduction of hormone binding. Moreover, the interaction of CHIP with Hsp70 may lead to an influence on SR at early stages of the folding cycle, similarly to TPR2 [34]. AR may be a special case, as CHIP interacts not only via Hsp90 with the LBD of this receptor, but also via its C-terminus with a conserved motif at the N-terminus of the receptor [17].

Increasing or reducing the levels of TPR2 has been shown to reduce the activity of GR and PR [34], [35], while other steroid receptors had not been analyzed before. In our experiments, increased levels of TPR2 resulted in a strong reduction of the activity of all SR, in contrast to the other investigated TPR proteins, which exhibited at least some selectivity in their action on SR. Our finding of strong interaction of TPR2 with Hsp70, but only moderate interaction with Hsp90 in comparison with other TPR proteins, supports the hypothesis that TPR2 acts by interference at early stages of the SR folding cycle [34], [35]. Furthermore, TPR2 still displayed considerable inhibitory activity at saturating conditions of hormone. Thus, TPR2 most likely operates through mechanisms in addition to reducing hormone binding affinity [34].

For XAP2, a moderate interaction with Hsp90 has been found before [37], [74], but there were no reports on incorporation into SR heterocomplexes. We reveal here the potential of XAP2 to interact with SR. This leads to a differential impact on the transcriptional activity of the receptors, with the strongest effects observed for GR and PR, while MR displayed little reaction to the presence of XAP2. XAP2 also interacts with other receptors, such as AhR [74], peroxisome proliferator-activated receptor α [75] and thyroid hormone receptor β1 [76]. These interactions go along with an inhibition of the transcriptional activity of PPARα, and a stimulation of AhR and THRβ1. XAP2 also affects nuclear translocation of AhR [77], [78] and GR [37].

FKBP51 and FKBP52 are the most intensely investigated TPR cofactors of steroid receptors. In particular for GR, important insight was gained from experiments in yeast, that characterised FKBP52 as stimulatory GR cofactor, while FKBP51 had no effect [18]. Studies in mammalian cells reported a strong inhibitory action of FKBP51 on GR, while over-expression of FKBP52 had no effect [20], [21], [79], [80]. In at least some mammalian cells, a positive effect of FKBP52 on AR- and GR-signaling has been observed [23], [24], [81]. Gene knock-out studies in mice revealed an essential influence of FKBP52 on AR- and PR-related physiological processes, while ablation of the FKBP51 gene did not result in an overt phenotype [24]–[27]. Very recently, a stimulatory effect of FKBP51 on AR has been reported in prostate cancer cells [82], [83]. We have obtained preliminary evidence that this may be a cell-type-specific effect (data not shown).

In the study presented here, FKBP51 and FKBP52 exhibited divergent effects on the transcriptional activities of GR, MR, PR and AR. Consistent with a previous report on GR [84], we also observed a stronger incorporation of FKBP51 in SR heterocomplexes than of FKBP52. At the same time, the interaction of both proteins with Hsp90 was comparable. Thus, the interaction with Hsp90 is not the sole determinant for the efficiency of integration into SR-heterocomplexes. Our interaction analyses further revealed a higher abundance of p23 in Hsp90 complexes with FKBP51 than in Hsp90 complexes with FKBP52. This may be explained by the possibly different interaction surfaces of Hsp90 that are engaged in binding these two immunophilins. FKBP52, in addition to the classic C-terminal MEEVD motif, recognises amino acids at the ATP binding pocket [85], which may impinge on p23 interaction. It should be noted, though, that binding of FKBP51 to this N-terminal site has not been tested yet [85]. Whatever the explanation is for the differential recruitment of p23, the increased presence of p23 may be related to the inhibitory action of FKBP51 on GR [86]. In addition, the recruitment of Hsp70 by FKBP51, albeit minor, could also add to the differential action of FKBP51 and FKBP52, as Hsp70 is absent in mature receptor heterocomplexes.

Our experiments revealed an inhibitory effect of PP5 that was most pronounced in the case of GR. Previous studies examining the effect of PP5 on GR produced partly inconsistent results. Expression of the TPR domain of PP5 in CV-1 cells abolished GR-dependent transcription [87], like probably the over-expression of any functional TPR-domain would do. On the other hand, over-expression of the PP5 TPR domain slightly stimulated the transactivation of ERα and ERβ [31], which reinforces the notion that the ERs differ in their TPR-protein dependency from the other SRs. Expression of full-length PP5 inhibited ERα and ERβ, probably via dephosphorylation of ER [31]. Knock-down of PP5 increased GR-dependent reporter gene activity in one study [30], while another study discovered a reduction of endogenous mRNA levels of GR-dependent genes [32]. In yeast, no effect of PP5 on GR was observed and PP5 was unable to compete with FKBP52 to decrease the GR-stimulation of this protein [18]. The inactivity of PP5 in yeast, which is insufficiently endowed with TPR-cofactors positive for GR, is compatible with our observations of the inhibitory action of PP5 in mammalian cells. Another study in yeast reported a positive effect of the PP5 yeast homologue Ppt1 on GR function, possibly due to removal of chaperone-inhibitory phosphates on Hsp90 [33]. It is not known whether this seeming discrepancy can be explained by differences between PP5 and its yeast homologue Ppt1, or by differences in GR regulation between yeast and mammalian cells in general.

The effects of the TPR proteins on SR observed here were significantly attenuated by saturating concentrations of hormone. This is consistent with an effect on hormone binding affinity. Different laboratories including ours provided evidence that later steps in steroid signal transduction are also affected by TPR cofactors, for example nuclear translocation [21], [88], [89] and dynamics of intranuclear mobility [90], which requires future experiments to clarify their relative contributions. Our results substantiate the concept that a delicate balance of TPR cofactors governs SR activity in a given cell or tissue, probably in a combinatorial fashion. The recent description of the N-terminal FKBP52 binding site on Hsp90 [85] opens the possibility for various combinations of two TPR proteins in the same SR heterocomplex. Alternatively, the dynamic assembly and disassembly of heterocomplexes may enable the sequential contribution of specific functions by the different TPR proteins.

## Materials and Methods

The MMTV-Luc reporter plasmid has been described previously [91]. ERE-Luc reporter plasmid and ERα and ERβ cDNA were a kind gift of Christian Behl (University Mainz). N-terminally HA-tagged ERα and ERβ were subcloned from ER cDNA into the pRK5-SV40 backbone. The plasmids expressing the N-terminally HA-tagged receptors GR, MR, PR and AR (pRK7 backbone) were kindly provided by Anke Hoffmann (MPI of Psychiatry, Munich) and the plasmid expressing Hsp90-FLAG by Len Neckers (NIH, Bethesda). The Gaussia-KDEL was constructed from a pCMV-GaussiaLuc1 plasmid (PJK) by linking the KDEL peptide sequence C-terminally via PCR, and subcloning into pRK5-SV40 backbone. TPR-proteins were all expressed as C-terminal FLAG-fusions in the pRK5-SV40 vector. The cDNA of CHIP was provided by Cam Patterson (University of North Carolina), of Cyp40 and TPR2 by Ulrich Hart (MPI of Biochemistry, Martinsried), of PP5 by Michael Chinkers (University of South Alabama). The plasmids expressing the FKBP51 and FKBP52 FLAG-fusions and the untagged FKBP52 in pRK5-SV40 were described previously [21]. The XAP2 plasmid is described in [37]. All plasmids were verified by sequencing. Primer sequences and cloning details are available upon request.

### Cell culture, transfection and reporter gene assay

Mouse embryonic fibroblasts (Marc Cox and David Smith, Mayo Clinic Scottsdale, Arizona, USA), human neuroblastoma SK-N-MC (ATTC HTB-10) and HEK-293 (ATTC CRL-1573) cells were cultured under conditions described previously [92], [93]. For the MMTV-luc reporter gene assay, cells were seeded in 96 well plates (SK-N-MC 30,000 cells/well; MEF 10,000 cells/well) in medium containing 10% charcoal-stripped, steroid-free serum and cultured for 24 h before transfection using ExGen (Fermentas) as described by the manufacturer. Unless indicated otherwise, the amounts of transfected plasmids per well were 60 ng of steroid responsive luciferase reporter plasmid MMTV-Luc, 5–7.5 ng of Gaussia-KDEL expression vector as control plasmid, 25 ng of plasmids expressing HA-tagged steroid hormone receptors (mGR in case of MEF cells) and up to 300 ng of plasmids expressing TPR-domain containing cofactors (not exceeding 200 ng per single TPR-protein expression plasmid). If needed, empty expression vector was added to the reaction to equal the total amount of plasmid in all transfections. 24 h after transfection, cells were cultured in fresh medium supplemented with hormone as indicated or ethanol as control for 24 h. To measure reporter gene activity cells were washed once with PBS and lysed in 50 µl passive lysis buffer (0.2% Triton X-100, 100 mM K_2_HPO_4_/KH_2_PO_4_ pH 7.8). Firefly and Gaussia luciferase activities were measured in the same aliquot using an automatic luminometer equipped with an injector device (Victor III, Wallac and Tristar, Berthold). Firefly activity was measured first by adding 50 µl Firefly substrate solution (3 mM MgCl_2_, 2.4 mM ATP, 120 µM D-Luciferin) to 10 µl lysate in black microtiter plates. By adding 50 µl Gaussia substrate solution (1.1 M NaCl, 2.2 mM Na_2_EDTA, 0.22 M K_2_H PO_4_/KH_2_PO_4_, pH 5.1, 0.44 mg/ml BSA, Coelenterazine 3 µg/ml) the firefly reaction was quenched and Gaussia luminescence was measured after a 5 s delay. Firefly activity data represent the ratio of background corrected Firefly to Gaussia luminescence values. The fold stimulation reached at saturating concentrations of hormone was for GR about 1000, which is in the range of previous publications [65], [91], MR 3.7, PR 970, AR 6, ERα 4, and ERβ 6.3. To compare the effects of co-expressed TPR proteins, the stimulation in the absence of the TPR protein was set to 100, and the stimulation in the presence of co-expressed TPR protein was referred to this value.

To check expression of receptors and TPR-proteins replicate lysates were pooled, briefly sonicated and cleared by centrifugation. Alternatively receptors and TPR-proteins were coexpressed in 6 well plates with the same receptor to TPR-protein ratios as for the 96 well plates. To this end, SK-N-MC cells were seeded in 6 well plates (500,000 cells/well) in medium containing 10% steroid-free serum and cultured for 24 before transfection of 0.25 µg HA-tagged steroid hormone receptors and corresponding amounts of plasmids expressing TPR-proteins per well using ExGen (Fermentas) as described by the manufacturer. If needed, empty expression vector was added to the reaction to equal the total amount of plasmid in all transfections. Cells were cultured as for the reporter gene assay and lysed in buffer containing 20 mM Tris-HCl pH 6.8, 0.67% SDS, 3.3% Sacharose completed with Protease Inhibitor Cocktail (Sigma), briefly sonicated and cleared by centrifugation. Lysates were analyzed by SDS-PAGE followed by immunoblot.

### Statistical Analysis

To improve our understanding of the effects of various TPR proteins on steroid receptor mediated gene transcription, we performed one sample t-tests to evaluate the significance of difference of the hormone-stimulated activity of the receptor in the presence versus absence of coexpressed TPR protein. Significance values were corrected according to the Bonferroni procedure. The most pronounced differences with a significance level of p≤0.001 are labelled in figures 3, 4 and 12 (*).

### Immunoblot

Immunoblot detection of proteins was performed largely as described [94]. Briefly, proteins were transferred from SDS gels to a nitrocellulose membrane (Schleicher & Schuell, GmbH). Non-specific binding to membrane was blocked by 5% nonfat milk in Tris-buffered saline supplemented with 0.1% Tween-20, and then one of the following specific primary antibodies were added: Actin (I-19, Santa-Cruz), FLAG tag-HRP (M2, Sigma); hemagglutinin tag-HRP (Roche Applied Science); p23 (ABR), FKBP52 (Anti-FKBP59, Stressgen), CHIP (PC711, Calbiochem), Cyp40 (ABR, PA3-022), FKBP51 (F14, Santa Cruz), XAP2 (ARA9, NB100-127, Novus Biologicals), TPR2 (kind gift of Ulrich Hartl), PP5/PPT (BD Biosciences). Signals were visualized by appropriate secondary antibodies conjugated to horseradish peroxidase and the ECL system (Millipore, Billerica, USA) and documented on X-ray film.

### Immunoprecipitation

For immunoprecipitation of FLAG-tagged TPR proteins or HA-tagged steroid receptors, HEK-293 cells were transfected with 2–10 µg of a plasmid expressing a TPR protein (amounts were adjusted to ensure comparable expression levels) and 5 µg of a plasmid expressing a steroid receptor. For Hsp90 precipitations, 10 µg of FLAG-tagged Hsp90 expression plasmid were transfected together with 10 µg of FKBP52 plasmid. HEK 293 cells were chosen, because they efficiently expressed the proteins and showed the same results in reporter gene assays as SK-N-MC cells. Transfection was performed by electroporation of one confluent 10 cm (60 cm^2^) dish (∼5×10^6^ cells) using a GenePulser (Bio-Rad, USA) at 350 V/700 µF in 400 µl of electroporation buffer (50 mM K_2_HPO_4_/KH_2_PO_4_, 20 mM KAc, pH 7.35, 25 mM MgSO_4_). Electroporated cells were replated in fresh medium containing 10% steroid-free serum containing medium and cultured for 3 days. Cells were harvested in cold PBS and lysed by resuspension in Lysis-Buffer A′ (130 mM NaCl, 20 mM Na_2_MoO_4_, 1 mM EDTA, 20 mM Tris-HCl pH 7.5, 10% Glycerol, 0.5% Triton X-100, completed with Protease Inhibtor cocktail, Sigma) for FLAG-TPR protein and receptor HA-IP, or in Hsp90 Lysis Buffer (20 mM Tris-HCl pH 7.5, 50 mM NaCl, 20 mM Na_2_MoO_4_, 1 mM EDTA, 1 mM EGTA, 0.1% NP-40, 10% Glycerol, 0.5 mM DTT, completed with Protease Inhibitor cocktail, Sigma and Phosphatase Inhibitor cocktail, Roche) for the Hsp90 FLAG-IPs, followed by brief sonication (Branson Cell Disruptor B15, 3×5 s, output 3) and incubation on ice for 1 h. The lysate was cleared by centrifugation (10 min, 25.000 rcf, 4°C) and the protein concentration was determined. 1–2 mg of lysate was incubated overnight at 4°C with the anti-FLAG M2 agarose affinity resin (Sigma) or with anti-HA agarose affinity resin (Sigma), respectively. FLAG-beads (30 µl slurry) were treated as recommended by the manufacturer. The next day, the beads were washed 3 times with Lysis Buffer without detergent and samples were eluted with 70 µl of 1× FLAG-peptide solution (Sigma, 100–200 µg/ml) or HA-peptide solution (Sigma, 100 µg/ml), respectively, in 1× Tris-buffered saline (150 mM NaCl, 10 mM Tris-HCl pH 7.0).

For analysis of the (co)precipitated proteins, 5–15 µg of the cell lysates or 25 µl of the immunoprecipitates were separated by SDS-PAGE under denaturing conditions. Coomassie staining was used for detection of immunoprecipitated TPR-proteins and coimmunoprecipitated Hsp90 and Hsp70 in the FLAG-IP. For all other detections immunoblots were used, i.e. the (co)precipitated steroid receptors in the FLAG- and HA-IP, co-precipitated FLAG tagged TPR-proteins in the HA-IP, and p23 in the FLAG-IP. To analyze relative binding, the signals were subjected to densitometry. The coomassie stained gels or films were scanned at 16 bit with a calibrated densitometer (GS800, Bio-Rad, USA) and analyzed with the Kodak 1D Image Analysis software.

To calculate the relative binding of co-precipitated proteins we proceeded as follows: For relative binding of the receptors to the precipitated TPR-proteins (FLAG-IP) the HA-immunoblot signals of the eluates were first normalized with the Coomassie density signals of the precipitated TPR proteins. To be able to compare results between different experiments, we calculated these data to represent relative receptor binding among the TPR proteins. To this end, the normalized receptor (HA-IB) signal for each TPR protein was divided by the mean of the normalized receptor signals of all TPR proteins in each experiment. These ratios could then be averaged throughout the different experiments.

Conversely, to calculate the relative binding of the associated TPR proteins to the precipitated receptors (HA-IP), the FLAG-immunoblot signals of the HA-IP eluates were normalized first with the HA-immunoblot signals of the HA-IP eluates (to correct for variations in precipitation efficiencies of the receptors), and second to the FLAG-immunoblot signals of the lysate (to correct for differences in TPR protein expression). To calculate the mean of different experiments, like for the FLAG-IP, the normalized signals of each TPR protein were represented in reference to the relative binding of the other TPR proteins. Because of variabilities of the HA-IPs, the relative binding of each TPR protein were not normalized to the mean of all TPR proteins, but for each receptor to the mean of a subset of TPR proteins. The subset of TPR proteins used to calculate the mean binding in different experiments are displayed in the figure legends of each receptor.

To analyze the relative binding of Hsp90, the Hsp90 (FLAG-IP)- Coomassie signals were normalized to the Coomassie signals of the precipitated TPR proteins and this relative binding was used to calculate the mean binding in different experiments.

To analyze relative Hsp70 binding to TPR proteins (FLAG-IP), first the Hsp70 signal of the control reaction ( = background Hsp70 binding) was subtracted from the Hsp70 coomassie signals of each TPR protein, and these values were then normalized to the coomassie signals of the precipitated TPR proteins. Slightly negative values were considered as no binding and set to zero. This relative binding was used to calculate the mean binding in different experiments. To analyze significant binding of Hsp70 to FKBP51, a two tailed heteroscedastic students t-test were applied.
